# Bacterial Diversity in Two Neonatal Intensive Care Units (NICUs)

**DOI:** 10.1371/journal.pone.0054703

**Published:** 2013-01-23

**Authors:** Krissi M. Hewitt, Frank L. Mannino, Antonio Gonzalez, John H. Chase, J. Gregory Caporaso, Rob Knight, Scott T. Kelley

**Affiliations:** 1 Department of Biology, San Diego State University, San Diego, California, United States of America; 2 University of San Diego Medical Center, San Diego, California, United States of America; 3 Department of Computer Science, University of Colorado at Boulder, Boulder, Colorado, United States of America; 4 Department of Biological Sciences, Northern Arizona University, Flagstaff, Arizona, United States of America; 5 Department of Computer Science, Northern Arizona University, Flagstaff, Arizona, United States of America; 6 Institute for Genomics and Systems Biology, Argonne National Laboratory, Argonne, Illinois, United States of America; 7 Department of Chemistry and Biochemistry, University of Colorado at Boulder, Boulder, Colorado, United States of America; 8 Howard Hughes Medical Institute, Boulder, Colorado, United States of America; Institute for Genome Sciences, University of Maryland, United States of America

## Abstract

Infants in Neonatal Intensive Care Units (NICUs) are particularly susceptible to opportunistic infection. Infected infants have high mortality rates, and survivors often suffer life-long neurological disorders. The causes of many NICU infections go undiagnosed, and there is debate as to the importance of inanimate hospital environments (IHEs) in the spread of infections. We used culture-independent next-generation sequencing to survey bacterial diversity in two San Diego NICUs and to track the sources of microbes in these environments. Thirty IHE samples were collected from two Level-Three NICU facilities. We extracted DNA from these samples and amplified the bacterial small subunit (16S) ribosomal RNA gene sequence using ‘universal’ barcoded primers. The purified PCR products were pooled into a single reaction for pyrosequencing, and the data were analyzed using QIIME. On average, we detected 93+/−39 (mean +/− standard deviation) bacterial genera per sample in NICU IHEs. Many of the bacterial genera included known opportunistic pathogens, and many were skin-associated (e.g., *Propionibacterium*). In one NICU, we also detected fecal coliform bacteria (Enterobacteriales) in a high proportion of the surface samples. Comparison of these NICU-derived sequences to previously published high-throughput 16S rRNA amplicon studies of other indoor environments (offices, restrooms and healthcare facilities), as well as human- and soil-associated environments, found the majority of the NICU samples to be similar to typical building surface and air samples, with the notable exception of the IHEs which were dominated by Enterobacteriaceae. Our findings provide evidence that NICU IHEs harbor a high diversity of human-associated bacteria and demonstrate the potential utility of molecular methods for identifying and tracking bacterial diversity in NICUs.

## Introduction

Low Birth Weight and Extremely Low Birth Weight (ELBW) infants in Neonatal Intensive Care Units (NICUs) are typically immunocompromised, and therefore susceptible to hospital-acquired infections [1], [2]. Infants admitted to NICUs, especially ones who have undergone surgery or have congenital abnormalities, are also often at high risk for developing nosocomial infections [2], [3]. An analysis of ELBW infant registry data from 1993–2001 by Stoll *et al.* (2004) illustrated the problems of NICU-acquired infections. This study found high infection rates, high mortality rates, and significantly increased risks of developing severe neuro-developmental disorders among infection survivors [4]. Specifically, Stoll *et al.* found that 65% (3932/6093) of ELBW infants contracted at least one infection during hospitalization; 38% had sepsis; 27% died from a hospital infection; surviving infected ELBW infants were significantly more likely than surviving uninfected infants to have severe neuro-developmental disorders; and 25% of ELBW infants had clinical manifestations of bacterial infection but were negative for culture growth.

One of the biggest difficulties in preventing NICU and Hospital-Acquired Infections (HAIs) is understanding the sources of the infectious agents and the routes of transmission. While culture-based methods are used to identify many infectious agents post-infection, it is not feasible to detect these a priori using culture-based techniques. The infectious bacteria may come from many different sources (e.g., individuals in the hospital, on surfaces, or on equipment), and even if it were possible to culture samples from all of these sources, culturing may still fail if growth conditions are not known, because a particular microbe grows very slowly (e.g., *Mycobacteria*), or samples were poorly handled.

Culture-independent methods based on amplification and sequencing of 16S rRNA genes allow identification of thousands of different bacteria in a single sample [5], [6], [7] when combined with high-throughput DNA sequencing, and hundreds of samples can be multiplexed simultaneously [7], [8]. In this study, we used high-throughput sequencing to investigate the diversity of bacteria found on inanimate hospital environments (IHEs; e.g., surfaces and equipment) in NICUs. While DNA sequencing methods cannot verify the current viability of particular microorganisms, they can determine the typical patterns and sources of surface microbial diversity. Although the importance of IHEs in spreading infections has been controversial [9], studies have shown that nosocomial pathogens can persist in a viable state for months on IHEs, and that contaminated rooms are a significant risk factor for infection [9], [10]. More recently, studies have shown that patients exposed to a contaminated environment are more likely to contract nosocomial pathogens [11], [12], [13], and that cleaning improvements can reduce infection rates of certain pathogens [14], [15], [16]. Our goal was to comprehensively characterize the microbial diversity of IHEs in NICUs, with a focus on commonly touched surfaces. We also compared our results to those of other indoor settings using similar molecular methods. Our results paint a broad picture of the source and extent of bacterial diversity on NICU IHEs.

## Methods

### Sample Collection

Samples were collected from two different large Level 3 Neonatal Intensive Care Units in San Diego, CA: one collection time in January 2009 (NICU1) and one collection time in February 2009 (NICU2). (The identities of the specific facilities were kept anonymous at the request of the medical staff.) Table 1 lists the surfaces sampled inside of the two facilities. Surface types sampled within the NICUs were chosen based on advice from the NICU medical personnel. We focused our sampling efforts primarily on surfaces that were likely to be touched before handling an infant. We also included less frequently touched surfaces, such as the inside of incubators and sink counters away from handles. Environmental samples were obtained with dual tip sterile cotton swabs (BBL CultureSwab™, catalog # 220135, Becton Dickinson, Sparks, MD). On flat surfaces (e.g., incubator plastic, counters and touch screens), approximately 12 cm^2^ of each surface was swabbed. Handles were swabbed in their entirety, and we swabbed a total of 9 keys on each of the keyboards and button pads. After sampling, the swab was immediately transported back to the lab on ice and stored at −80**°**C until DNA extraction.

**Table 1:** Locations of surface sampling performed in two NICU facilities.

NICU	Station	Surface	NICU	Station	Surface
NICU 1	Baby Bedside	Diaper Scale	NICU 2	Baby Bedside	Diaper Scale
		Drawer Handle			Plastic Side
		Touch Screen			Plastic Side
	Door Button	Button Surface			Drawer Handle
		Button Surface			Touch Screen
	Incubator	Incubator		Door Button	Button Surface
		Keyboard			Button Surface
		Drawer Handle		Incubator	Inside Incubator
		Turn Handle			Keyboard
	Pyxus	Keyboard			Drawer Handle
	Sink	Sink Counter			Turn Handle
	Weigh Cart	Drawer Handle		Pyxus	Keyboard
		Drawer Handle		Sink	Cabinet Handle
					Counter By Sink
					Sink Handle
				Weigh Cart	Drawer Handle
					Drawer Handle

### DNA Extraction and PCR

Prior to DNA extraction, the cotton from the swab was removed using a flame-sterilized razor blade and the cotton threads were deposited into a lysozyme reaction mixture. The DNA extractions for all 30 swabs from surfaces in both NICUs and two negative controls were conducted at the same time. The reaction mixture had a total volume of 200 µl and included the following final concentrations: 20 M Tris, 2 mM EDTA (pH 8.0), 1.2% P40 detergent, 20 mg ml^−1^ lysozyme, and 0.2 µm filtered sterile water (Sigma Chemical Co., St. Louis, MO). Samples were incubated in a 37°C water bath for thirty minutes. Next, Proteinase K (DNeasy Tissue Kit, Qiagen Corporation, Valencia, CA) and AL Buffer (DNeasy Tissue Kit, Qiagen Corporation, Valencia, CA) were added to the tubes and gently mixed. Samples were incubated in a 70°C water bath for 10 min. All samples were subjected to purification using the DNeasy Tissue Kit. Following extraction, the DNA was quantified using a NanoDrop ND-1000 Spectrophotometer (NanoDrop Technologies, Willmington, DE).

PCR reactions were performed in small lots (six plus positive and negative extraction and PCR controls) to reduce the possibility of laboratory contamination. Barcoded PCR amplification was performed with the widely used 27F and 338R 16S rRNA “universal” bacterial primers. The primers flank the highly variable V2 region of the 16S rRNA gene sequence that is taxonomically informative across most of the bacteria. The barcodes allowed us to pool the PCR products from all samples into one 454 sequencing run. The forward primer constructs consist of a short adapter sequence necessary for the pyrosequencing reaction, the unique 12-base DNA “barcode” encoded with Golay codes [17], and the universal primer sequence. PCR was carried out in a total reaction volume of 50 µl including 1 µl (approx. 10 ng µl^−1^) of sample DNA as template, each deoxynucleoside triphosphate at 400 µM, 1.65 mM MgCl_2,_ 5 µl 10× buffer (10× concentration: 500 mM 1 M KCl, 100 mM 1 M Tris HCl pH 8.4, 1% Triton-X), each primer at 1 µM, and 1 µl of REDTAQ™ DNA polymerase (1 unit µl^−1^; Sigma-Aldrich Inc., St. Louis, MO). Thirty cycles of PCR amplification were performed for the environmental swab samples. We used the lowest number of cycles that yielded a visible band on an agarose gel in order to minimize differential amplification of sequences and production of chimeric sequences. All PCR cycles included an initial denaturation step at 94°C for 1 min, an annealing step at 55°C for 45 sec and an extension step at 72°C for 1.5 min. The amplification cycles were preceded by a one-time denaturing step at 94°C for 5 min prior to the first cycle and included a final 72°C extension for 10 min to ensure complete extension.

### Sequencing

Individual barcoded PCR products were purified using the AMPure purification kit (Agenourt, Beverly) following the manufacturer’s protocol. After Ampure purification every sample was quantified on an Agilent 2100 Bioanalyzer. All samples were diluted down to 2×10^−5^ moles/µL (50 µL volume) and were then pooled with a total combined concentration of 2×10^−5^ moles/µL (100 µL total volume). PCR purification, dilutions and pyrosequencing on a 454 Life Sciences FLX Genome Sequencer were all conducted by the core facility at the University of South Carolina (Environmental Genomics Core Facility).

### Computational and Statistical Analyses

The barcoded pyrosequencing data was analyzed using the QIIME database (www.microbio.me/qiime), QIIME [18] version 1.5.0-dev, SitePainter 1.1 [19] and SourceTracker 0.9.4 [20].

**Figure 1:**
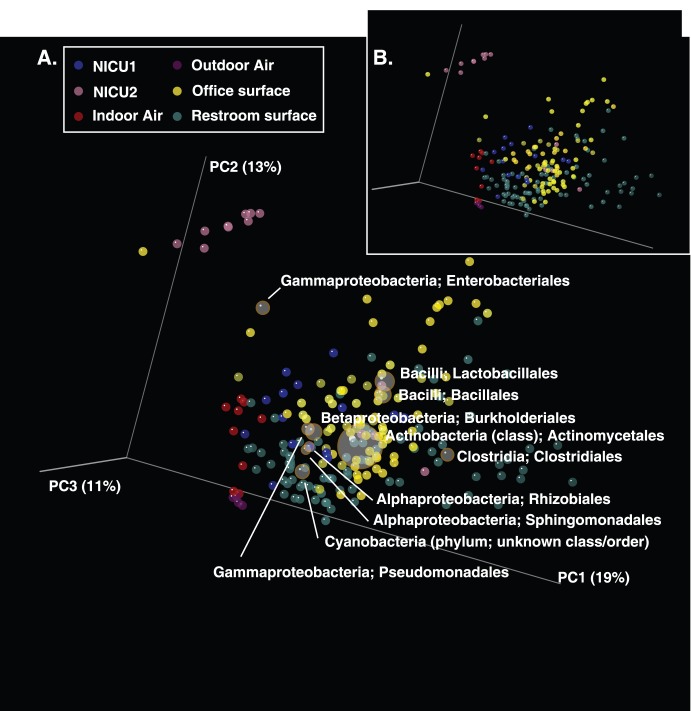
PCoA analysis of NICU samples and previously published indoor studies. PCoA of pair-wise weighted UniFrac distances (see Methods) both with biplots that include taxonomy (A), and without biplots (B). The different colored points indicate the various indoor sampling environments Most of the NICU samples cluster with other indoor surface samples, except for nine NICU1 samples in the top left which cluster with a single office surface sample. Order-level taxonomy illustrates that the presence of Enterobacteriales contributes to the distinct clustering of these samples.

As part of the QIIME database processing pipeline, raw sequence data were split into samples by barcode, and low quality reads were filtered using the QIIME database’s default parameters. Each library was sub-sampled to an even sequencing depth of exactly 500 sequences per sample to mitigate biases arising from different depths of sequence across samples, and clustered into operational taxonomic units (OTUs) using a closed-reference OTU picking protocol at the 97% sequencing identity level using UCLUST [21] against the Greengenes database [22] pre-clustered at 97% sequence identity. The closed-reference OTU picking protocol was applied to allow direct comparison of these samples to samples amplified with different “universal” 16S rRNA PCR primers The taxonomy associated with each OTU was assigned as the taxonomy associated with the reference sequence defining the OTU, and the corresponding Greengenes tree was used to compute weighted UniFrac [23] distances between samples. Principal Coordinates Analysis (PCoA) was applied to summarize UniFrac distance matrices and generate biplots including taxa (Figure 1).

**Figure 2:**
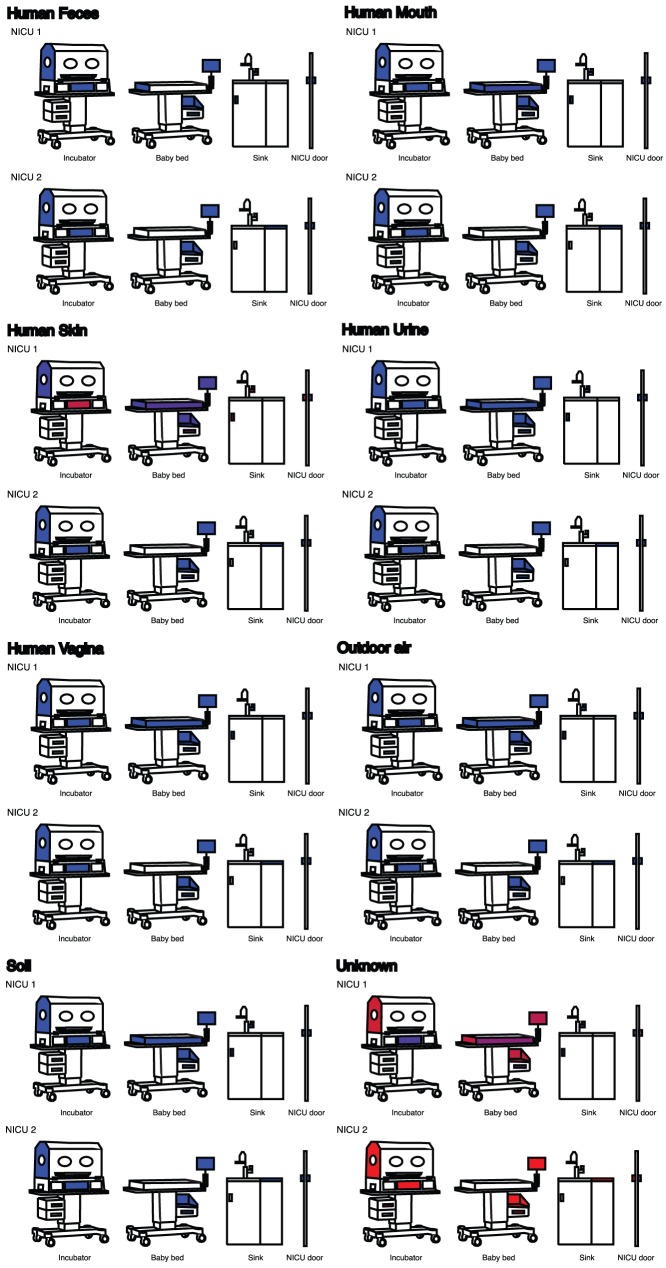
Likely sources of microbes in the two NICUs. SitePainter images display the results from SourceTracker. The NICU sites are colored on a heatmap scale, where blue indicates that low similarity between a sink and a source and red indicates high similarity between a sink and a source Many surfaces have microbial compositions that are not similar to any of the sources (represented by Unknown), while the handles of the drawers, door and faucet, and the keyboard of the incubator, resemble the communities of human skin.

As part of the QIIME database processing, a metaanalysis was performed to compare our two NICUs to data obtained in five previously published studies: office workspaces in three major cities [24]; restroom surfaces [25];, airborne bacteria in a health-care facility [26]; human-associated microbial communities from different body sites [27]; and diverse soil types [28]. All three studies followed similar protocols for DNA purification, 16S amplification and pyrosequencing but, several studies used different bacterial 16S primers than our NICU samples, requiring the use of a closed-reference OTU picking protocol. This protocol has been shown to be reliable for comparing sequence data generated on different sequencing platforms and from different primer pairs [29]. SourceTracker was applied treating the human-associated (skin, feces, mouth, vagina, and urine), outdoor air, and soil communities as sources and the NICU sites as sinks (Figure 2).

All sample metadata is providing in Supplementary Table S3 as a QIIME-compatible mapping file. We present the exact series of commands that were applied to perform our bioinformatics analyses as supplementary methods (Table S4) and the OTU tables are supplied in QIIME-compatible text (Table S5) and biom (Table S6) formats.

## Results

DNA extractions from all surface samples contained measurable quantities of bacterial DNA (4–10 ng/µl): negative extraction controls had no quantifiable DNA. All samples produced visible PCR products, and the negative PCR and DNA extraction controls were blank. The pyrosequencing of 30 surface swab samples yielded a total of 321,000 sequences with an average of 245bp (75.9 Mb of data). There were approximately 193,546 sequences after removal of the low quality reads and closed-reference OTU picking. Approximately 37,384 sequences were obtained from NICU1, averaging 2200 reads per surface, while 156,162 sequences were obtained from NICU2, averaging 12,012 reads per surface.

Table 2 lists the 17 different bacterial genera containing species with known opportunistic pathogens that were found commonly in both NICU facilities (although we emphasize that our techniques did not allow species-level identification). Supplementary tables S1 and S2 list of genera present an expansive list of bacterial genera and the final OTU tables output by QIIME are included as supplementary data. Figure 1 presents the results of a Principal Coordinate Analysis (PCoA) produced using QIIME and based on the pairwise weighted UniFrac distances between all the NICU samples, both including (Figure 1A) and not including (Figure 1B) biplots showing the prevalent taxa. Figure 2 presents the suspected sources of the microbial communities in the different NICU sites.

**Table 2:** Most common bacterial genera (with known opportunistic pathogens) found in both NICUs (see Tables S1 and S2 for full list).

Acinetobacter	Microbacterium
Actinomyces	Neisseria
Burkholderia	Pasteurella
Clostridium	Propionibacterium
Enterobacter	Pseudomonas
Flavimonas	Roseomonas
Flavobacterium	Staphylococcus
Gemella	Stenotrophomonas
Leclercia	Streptococcus

## Discussion

The culture-independent high-throughput sequencing methods employed in the NICU facilities revealed far more microbial diversity than previously revealed by culture-dependent or targeted molecular PCR analyses of NICU surfaces. Every surface we sampled was inhabited by tens to hundreds of bacterial genera, averaging approximately 100 bacterial genera per surface. These included genera containing many known opportunistic pathogens (Table 2), as well as abundant groups whose pathogenic potential and ability to resist antibiotic treatment are poorly understood (Table S1, S2). While we detected substantially more diversity with the 16S rRNA methods than typically found with culture-based methods, many of the genera in our study are commonly found in culture-based studies of hospital environments and specifically associated with Hospital-Acquired Infections (HAIs) in neonatal patients. Species of *Enterobacter*, *Neisseria*, *Pseudomonas*, *Streptococcus*, and *Staphylococcus* were found abundantly in both NICUs (Table 2). Members of these genera are known to cause nosocomial infections in infants [30], [31]. We also found evidence of other opportunistic pathogens that routinely cause nosocomial infections, including *Acinetobacter*, *Clostridium*, *Flavimonas*, *Flavobacterium*, *Fusobacterium*, *Gemella*, *LeClercia*, *Legionella*, *Pasteurella*, *Propionibacterium*, and *Stenotrophomonas* [32], [33]. We also observed a substantial number of organisms that are not readily cultured (Table S1, S2).

Our Principal Coordinates Analysis (PCoA) of the pairwise weighted UniFrac distances between samples in the NICUs found that nine of the NICU1 surfaces were easily separable from the rest of the surface samples from both NICU1 and NICU2 (top left of Figure 1A). Most of the NICU samples clustered with other indoor samples from office, healthcare centers and restrooms. However, PCoA showed that those nine NICU1 samples were clearly divergent from the rest of the samples (Figure 1, pink points). Many of the restroom surface samples (green points) were also quite different from indoor office surfaces (yellow) and air (indoor hospital air (red), outdoor hospital air (purple).

A closer inspection of the microbial diversity in the divergent NICU1 samples indicated that an excess of Enterobacteriaceae sequences was responsible for the divergence of these samples (Figure 1A). Members of the Enterobacteriaceae (e.g., *E. Coli*, *Klebsiella*, *Enterobacter*, *Salmonella*) commonly inhabit the digestive tract and can be found abundantly in feces. *E. coli*, *Klebsiella* and *Enterobacter* in particular are very well known hospital ICU pathogens that appear to spread and proliferate quite easily in hospitals [32], and many of whom appear to be developing multi-drug resistance [34], [35]. Another very consistent finding was the high proportion of bacterial genera associated with human skin, particularly *Propionibacterium*, which was one of the most common in both NICUs. We also found considerable proportions of *Corynebacterium*, *Lactobacillus, Staphylococcus*, and *Streptococcus*, all of which are very common on hand surfaces [17].

According to Flores et al. (2011), some restroom samples were either dominated by gut- (fecal), vagina- and soil-associated bacteria, while others were dominated by skin-associated bacteria. The majority of the office surfaces contained both skin and soil-associated bacteria [20] and clustered with the frequently hand-touched restroom surfaces (e.g., door and handle surfaces; Figure 1). SourceTracker shows the source of NICU surface microbes is often human skin to the exclusion of the other sources that were investigated here (Figure 2). Interestingly, we noticed that NICU1 surfaces seem to resemble human skin far more than NICU2 surfaces. We suspect that this might result from more recent cleaning of NICU2, but unfortunately we do not know when each room was last cleaned. These findings lend considerable weight to the notion that human hands are important vectors for transmitting bacteria in NICU facilities.

Our data provide evidence that NICUs harbor pools of diverse bacteria, that NICU diversity is similar to other indoor surface environments, and that human skin is a primary contributor indicating that hand transfer (touch) can move organisms through such settings. Future work in hospitals should attempt to integrate these molecular methods with long-term assessment of surface bacterial diversity and infection rates over time and record the cleaning schedule to investigate the rate at which skin microbes colonize IHEs.

One clear limitation to our study is that we cannot determine which of the microbes we identified were viable. Non-viable organisms cannot directly cause infection, although they may still contribute antibiotic resistance genes to the wider bacterial community. Numerous previous studies have shown the potential ability of many of the microbes detected by molecular methods to be viable, and it is generally easy to grow microbes from any given indoor surface. Setting aside the futility of trying to cultivate dozens or hundreds of different microbes from even a single sample, future work in this area would benefit from the combination of molecular and cultivation assays, increasingly rapid sequencing technologies, and perhaps the addition of molecular assays that simultaneously determine diversity and viability.

A drawback of this approach is the lack of taxonomic resolution at the strain level, which can be problematic for differentiating pathogens from their non-pathogenic close relatives. Short reads of the 16S rRNA obtained (as obtained with recent sequencing technologies) are effective at providing broad, genus-level characterization of microbial communities. Longer reads or different marker genes that provide strain-level resolution for taxonomic groups of interest (i.e., genes with higher rates of accumulated mutations and therefore more divergence between species and strains) will likely be necessary to accurately detect the presence of specific pathogenic bacterial species and strains.

As these high-throughput methods become cheaper and easier, and as the associated bioinformatics becomes more accessible, techniques such as those described here could be routinely applied in detecting or monitoring the spread of bacteria in NICUs. By detecting departures from “typical” NICU bacterial diversity, an early warning system for infectious agents could be developed. To achieve these goals, more data (including NICU surface time-series data) will need to be gathered to understand what normal bacterial diversity and temporal variability looks like on NICU surfaces. This information is essential to accurately identifying deviations from normality.

## Supporting Information

Table S1 **Raw sequence counts of bacterial genera found on NICU1 surfaces.** Identifications were made using the Ribosomal Database Project Classifier (Wang et al. 2007; see Methods). Genera with less than a combined total of 50 sequence matches were excluded from the table. Genera containing known opportunistic pathogens are highlighted in boldface.
(DOC)

Table S2 **Raw sequence counts of bacterial genera found on NICU 2 surfaces.** Genera containing known opportunistic pathogens are highlighted in boldface.
(DOC)

Table S3 **QIIME-compatible sample metadata mapping file.**
(TXT)

Table S4 **Supplementary Methods.**
(DOCX)

Table S5 **QIIME-compatible text-formatted OTU table.**
(GZ) Click here for additional data file.

Table S6 **QIIME-compatible biom–formatted OTU table.**
(GZ) Click here for additional data file.
