# Supplementary material for: Bacterial Diversity in Two Neonatal Intensive Care Units (NICUs)
Source: PLoS One. 2013 Jan 23;8(1):e54703. doi: 10.1371/journal.pone.0054703 (PMC3553055; doi:10.1371/journal.pone.0054703)
Supplement: Table S1 — Raw sequence counts of bacterial genera found on NICU1 surfaces. Identifications were made using the Ribosomal Database Project Classifier (Wang et al. 2007; see Methods). Genera with less than a combined total of 50 sequence matches were excluded from the table. Genera containing known opportunistic pathogens are highlighted in boldface. (DOC) [file pone.0054703.s001.doc]

**Table S1.** Raw sequence counts of bacterial genera found on NICU1 surfaces. Identifications were made using the Ribosomal Database Project Classifier (Wang et al. 2007; see Methods). Genera with less than a combined total of 50 sequence matches were excluded from the table. Genera containing known opportunistic pathogens are highlighted in boldface.

| **Genus** | **Door Buttons** | **Diaper Scale** | **Drawer Handles** | **Key- boards** | **Inside Incubator** | **Sink** | **Touch screen** | **Count Totals** |
| --- | --- | --- | --- | --- | --- | --- | --- | --- |
| *Acaricomes* | 31 | 10 | 176 | 109 | 24 | 26 | 28 | 404 |
| *Acidovorax* | 9 | 13 | 148 | 17 | 29 | 22 | 30 | 268 |
| ***Acinetobacter*** | **23** | **23** | **539** | **651** | **21** | **30** | **20** | **1307** |
| ***Actinomyces*** | **5** | **4** | **62** | **45** | **6** | **16** | **12** | **150** |
| *Algibacter* | 27 | 13 | 19 | 11 |  | 5 | 6 | 81 |
| *Anaerococcus* | 21 | 3 | 20 | 54 |  | 3 | 4 | 105 |
| *Arthrobacter* | 7 | 21 | 38 | 39 | 10 | 19 | 33 | 167 |
| *Asticcacaulis* | 28 |  | 32 | 17 |  | 11 | 4 | 92 |
| *Bacillus* | 2 | 18 | 67 | 24 | 13 | 6 | 17 | 147 |
| ***Bacteroides*** | **133** | **2** |  | **31** |  |  |  | **166** |
| *Beggiatoa* |  | 12 | 166 | 17 | 5 |  | 7 | 207 |
| *Blastobacter* | 1 | 3 | 50 | 15 | 1 | 4 | 6 | 80 |
| *Bradyrhizobium* | 104 | 14 | 280 | 45 | 24 | 19 | 11 | 497 |
| ***Burkholderia*** | **1468** | **123** | **4743** | **1326** | **116** | **115** | **163** | **8054** |
| *Caulobacter* | 6 | 11 | 76 | 44 | 14 | 6 | 3 | 160 |
| *Cetobacterium* | 31 | 8 | 85 | 10 | 3 | 5 | 5 | 147 |
| *Chryseobacterium* | 15 | 14 | 170 | 23 | 15 | 32 | 14 | 283 |
| *Cloacibacterium* | 10 | 1 | 67 | 4 |  | 3 | 2 | 87 |
| ***Clostridium*** | **1** | **1** | **45** | **5** | **15** | **1** | **1** | **69** |
| *Collimonas* | 1 | 6 | 36 | 9 | 4 | 2 | 14 | 72 |
| *Conexibacter* | 11 | 3 | 27 | 6 | 7 | 2 | 5 | 61 |
| *Coprococcus* | 2 | 3 | 27 | 7 | 4 | 7 | 4 | 54 |
| *Corynebacterium* | 136 | 175 | 1725 | 1272 | 174 | 211 | 269 | 3962 |
| *Delftia* | 54 | 38 | 252 | 37 | 10 | 19 | 25 | 435 |
| *Derxia* | 24 | 37 | 151 | 31 | 50 | 38 | 74 | 405 |
| *Devosia* | 1 |  | 21 | 17 | 3 | 1 | 7 | 50 |
| *Duganella* | 10 | 21 | 37 | 34 | 34 | 23 | 29 | 188 |
| *Enhydrobacter* | 20 | 5 | 56 | 25 | 5 | 9 | 3 | 123 |
| ***Enterobacter**** | **8292** | **9815** | **27600** | **9290** | **16902** | **15077** | **14410** | **101386** |
| *Enterovibrio* | 39 |  | 78 | 4 |  | 1 | 3 | 125 |
| *Erythromicrobium* | 2 |  | 67 | 16 | 4 | 6 | 2 | 97 |
| *Faecalibacterium* | 89 |  | 12 | 2 |  |  |  | 103 |
| *Finegoldia* | 17 | 6 | 16 | 37 |  |  |  | 76 |
| ***Flavimonas*** | **25** | **40** | **316** | **182** | **34** | **49** | **37** | **683** |
| ***Flavobacterium*** | **21** | **6** | **52** | **6** | **5** | **7** |  | **97** |
| *Fluviicola* | 12 | 2 | 30 | 10 | 1 | 4 |  | 59 |
| *Friedmanniella* | 2 | 2 | 4 | 15 | 13 | 8 | 9 | 53 |
| ***Fusobacterium*** | **21** |  | **40** | **34** | **9** | **13** | **8** | **125** |
| ***Gemella*** | **12** | **4** | **20** | **46** | **7** | **22** | **14** | **125** |
| *Geobacillus* |  | 5 | 64 | 9 | 6 | 10 | 6 | 100 |
| *Gp4* | 13 | 7 | 25 | 5 | 1 | 3 | 4 | 58 |
| *Gracilibacillus* |  |  | 53 |  | 1 | 1 |  | 55 |
| *Guggenheimella* | 26 | 1 | 44 | 6 | 3 |  |  | 80 |
| *Hymenobacter* | 11 | 15 | 32 | 22 | 23 | 32 | 23 | 158 |
| *Hyphomonas* | 2 | 13 | 29 | 3 | 4 | 7 | 4 | 62 |
| *Janthinobacterium* | 4 | 11 | 97 | 29 | 7 | 12 | 1 | 161 |
| *Lachnobacterium* | 127 |  |  |  |  | 1 | 2 | 130 |
| *Lachnospiraceae* | 79 |  | 3 | 15 | 2 | 1 |  | 100 |

**Supplementary Table S1.** (Continued)

| **Genus** | **Door Buttons** | **Diaper Scale** | **Drawer Handles** | **Key-boards** | **Inside Incubator** | **Sink** | **Touch screen** | **Count Totals** |
| --- | --- | --- | --- | --- | --- | --- | --- | --- |
| *Lactobacillus* | 837 | 2526 | 8542 | 1457 | 2671 | 3622 | 3483 | 23138 |
| ***Leclercia*** | **192** | **86** | **825** | **177** | **135** | **155** | **108** | **1678** |
| *Malikia* | 5 | 12 | 158 | 24 | 16 | 13 | 12 | 240 |
| *Marinithermus* | 1 |  | 45 | 1 | 1 | 2 | 14 | 64 |
| *Melissococcus* | 49 | 158 | 457 | 101 | 156 | 177 | 219 | 1317 |
| *Methylobacterium* | 25 | 24 | 149 | 47 | 27 | 28 | 26 | 326 |
| *Methylopila* | 6 | 1 | 15 | 1 | 14 | 17 | 7 | 61 |
| ***Microbacterium*** | **10** | **3** | **61** | **56** | **24** | **25** | **13** | **192** |
| *Microvirgula* | 14 | 9 | 109 | 25 | 5 | 12 | 23 | 197 |
| *Millisia* | 21 | 1 | 144 | 34 | 38 | 40 | 42 | 320 |
| *Mitsuaria* |  | 1 | 35 |  | 5 | 6 | 6 | 53 |
| ***Neisseria*** | **8** | **39** | **213** | **68** | **41** | **30** | **74** | **473** |
| *Niastella* | 12 | 2 | 144 | 8 | 6 | 7 | 10 | 189 |
| *Oligotropha* | 6 | 2 | 93 | 8 | 3 | 6 | 3 | 121 |
| *Oribacterium* | 3 | 5 | 31 | 2 | 3 | 14 | 2 | 60 |
| *Paenibacillus* | 21 |  | 19 | 8 | 1 | 2 | 7 | 58 |
| *Paracoccus* | 8 | 6 | 47 | 10 | 6 | 1 | 10 | 88 |
| *Paralactobacillus* | 5 | 43 | 146 | 32 | 34 | 31 | 42 | 333 |
| ***Pasteurella*** | **18** | **10** | **371** | **49** | **14** | **54** | **50** | **566** |
| *Pasteuriaceae* | 2 | 1 | 58 | 1 | 2 |  | 1 | 65 |
| *Paucisalibacillus* | 1 | 2 | 28 | 11 |  | 3 | 5 | 50 |
| *Peptoniphilus* | 5 | 6 | 88 | 40 | 2 | 4 | 4 | 149 |
| *Porphyromonas* | 19 | 2 | 190 | 20 | 18 | 6 | 5 | 260 |
| *Prevotella* | 22 | 4 | 70 | 42 | 5 | 6 | 12 | 161 |
| ***Propionibacterium*** | **1007** | **290** | **7353** | **2145** | **443** | **487** | **733** | **12458** |
| ***Pseudomonas*** | **5** | **3** | **29** | **6** | **19** | **3** | **13** | **78** |
| *Pseudonocardia* | 11 | 12 | 39 | 48 | 16 | 22 | 20 | 168 |
| *Pseudovibrio* |  |  |  | 291 |  |  |  | 291 |
| *Psychrobacter* | 8 | 20 | 203 | 22 | 27 | 17 | 26 | 323 |
| *Reinekea* | 2 | 7 | 43 | 9 | 11 | 6 | 11 | 89 |
| *Rhodobacter* | 15 | 18 | 57 | 37 | 28 | 32 | 44 | 231 |
| ***Roseomonas*** | **7** | **9** | **30** | **36** | **10** | **8** | **24** | **124** |
| *Ruminococcus* | 96 | 2 |  |  |  |  |  | 98 |
| *Salinibacterium* | 16 | 2 | 52 | 25 | 10 | 5 | 7 | 117 |
| *Salmonella* | 7 | 8 | 50 | 19 | 31 | 12 | 30 | 157 |
| *Sandaracinobacter* | 1839 | 188 | 5331 | 409 | 311 | 284 | 321 | 8683 |
| *Sphingobium* | 19 | 15 | 143 | 7 | 13 | 14 | 27 | 238 |
| *Sphingosinicella* | 30 | 22 | 167 | 60 | 28 | 35 | 57 | 399 |
| ***Staphylococcus*** | **232** | **198** | **2611** | **857** | **187** | **192** | **188** | **4465** |
| ***Stenotrophomonas*** | **19** | **8** | **95** | **32** | **28** | **22** | **22** | **226** |
| ***Streptococcus*** | **73** | **73** | **2054** | **588** | **89** | **137** | **440** | **3454** |
| *Terrimonas* | 16 | 8 | 39 | 7 | 3 | 11 | 26 | 110 |
| *Thalassomonas* | 6 | 9 | 25 | 4 | 5 | 6 | 2 | 57 |
| *Thiobacter* | 1 | 1 | 49 | 5 |  | 1 | 5 | 62 |
| *Veillonella* | 15 | 11 | 86 | 41 | 3 | 9 | 11 | 176 |
| ***Vibrio*** | **56** | **7** | **297** | **31** | **16** | **19** | **6** | **432** |
| *Xylella* |  | 9 | 51 | 4 | 6 | 9 | 2 | 81 |
| *Xylophilus* | 40 | 76 | 170 | 26 | 87 | 70 | 65 | 534 |

* Includes *Enterobacter* and other members of Enterobacteriaceae
