# Supplementary material for: Bacterial Diversity in Two Neonatal Intensive Care Units (NICUs)
Source: PLoS One. 2013 Jan 23;8(1):e54703. doi: 10.1371/journal.pone.0054703 (PMC3553055; doi:10.1371/journal.pone.0054703)
Supplement: Table S2 — Raw sequence counts of bacterial genera found on NICU 2 surfaces. Genera containing known opportunistic pathogens are highlighted in boldface. (DOC) [file pone.0054703.s002.doc]

**Table S2.** Raw sequence counts of bacterial genera found on NICU 2 surfaces. Genera containing known opportunistic pathogens are highlighted in boldface.

| **Genus** | **Door Buttons** | | | **Diaper Scale** | | **Drawer Handles** | **Key-**  **boards** | **Inside Incubator** | **Sink** | **Touch screen** | | **Count Totals** |
| --- | --- | --- | --- | --- | --- | --- | --- | --- | --- | --- | --- | --- |
| *Acaricomes* |  | | | 8 | | 41 | 49 |  | 17 |  | | 115 |
| *Acidovorax* | 16 | | | 4 | | 49 | 19 | 5 | 3 | 30 | | 126 |
| ***Acinetobacter*** | **91** | | | **364** | | **96** | **38** | **30** | **22** | **96** | | **737** |
| ***Actinomyces*** | **29** | | |  | | **27** | **37** |  | **15** |  | | **108** |
| *Actinoplanes* |  | | | 1 | | 33 | 9 |  | 6 | 28 | | 77 |
| *Algibacter* | 41 | | | 4 | | 9 | 15 | 39 | 4 | 33 | | 145 |
| *Anaerobacter* | 8 | | | 1 | | 6 | 7 | 28 | 1 |  | | 51 |
| *Anaerococcus* | 30 | | |  | | 56 | 22 |  | 24 |  | | 132 |
| *Anaeromyxobacter* |  | | |  | | 41 |  |  | 4 | 1 | | 46 |
| *Arthrobacter* | 5 | | | 12 | | 44 | 10 |  | 55 | 20 | | 146 |
| *Aurantimonas* |  | | |  | |  | 8 |  | 51 |  | | 59 |
| *Bacillus* |  | | | 232 | | 1 | 6 | 10 | 19 |  | | 268 |
| ***Bacteroides*** |  | | | **69** | | **12** | **2** |  | **2** |  | | **85** |
| *Beggiatoa* | 32 | | | 13 | | 64 | 16 | 54 | 10 | 242 | | 431 |
| *Blastobacter* |  | | | 19 | |  | 4 |  | 45 | 1 | | 69 |
| *Bradyrhizobium* | 211 | | | 37 | | 157 | 80 | 112 | 70 | 590 | | 1257 |
| *Brevundimonas* |  | | | 38 | |  | 3 |  | 19 | 3 | | 63 |
| ***Burkholderia*** | **3160** | | | **790** | | **2796** | **1145** | **1254** | **869** | **3449** | | **13463** |
| *Caulobacter* | 52 | | |  | | 70 | 19 | 6 | 113 | 103 | | 363 |
| *Cetobacterium* | 63 | | | 15 | | 24 | 12 | 4 | 6 | 26 | | 150 |
| *Chitinophaga* | 26 | | |  | | 4 |  | 35 |  |  | | 65 |
| *Chryseobacterium* |  | | | 15 | | 35 | 27 | 1 | 18 | 19 | | 115 |
| *Corynebacterium* | 219 | | | 133 | | 1321 | 516 |  | 114 |  | | 2303 |
| *Delftia* | 172 | | | 55 | | 182 | 51 | 159 | 30 | 183 | | 832 |
| *Derxia* | 4 | | | 7 | | 10 | 5 | 4 | 6 | 37 | | 73 |
| *Enhydrobacter* |  | | | 6 | | 19 | 7 |  | 870 |  | | 902 |
| ***Enterobacter*** | | **44** | **37** | | **755** | | **4** |  | **5** | | **3** | **848** |
| *Enterovibrio* | 74 | | | 5 | | 79 | 13 | 12 | 18 | 123 | | 324 |
| *Erythrobacter* |  | | | 2 | |  | 5 |  | 105 |  | | 112 |
| *Finegoldia* | 16 | | |  | | 19 | 7 | 45 |  |  | | 87 |
| ***Flavimonas*** | **150** | | | **726** | | **320** | **25** | **62** | **33** | **229** | | **1545** |
| ***Flavobacterium*** | **13** | | | **3** | | **34** | **25** | **49** | **11** | **45** | | **180** |
| *Flectobacillus* | 50 | | |  | | 10 |  | 14 |  |  | | 74 |
| *Fluviicola* | 4 | | |  | | 11 | 7 | 5 | 1 | 17 | | 45 |
| ***Gemella*** | **29** | | | **24** | | **13** | **11** |  | **5** | **19** | | **101** |
| *Geobacillus* | 34 | | |  | |  | 6 |  |  |  | | 40 |
| *Guggenheimella* | 22 | | |  | | 28 | 9 | 28 | 4 | 105 | | 196 |
| *Halomonas* | 32 | | |  | |  | 1 | 3 | 5 | 46 | | 87 |
| *Janthinobacterium* | 22 | | |  | | 15 | 1 |  | 8 |  | | 46 |
| *Lachnospiraceae* | 12 | | | 73 | | 9 | 24 |  | 3 |  | | 121 |
| *Lactobacillus* |  | | | 9 | | 75 | 7 |  | 16 | 2 | | 109 |
| ***Leclercia*** | **346** | | | **913** | | **481** | **77** | **156** | **78** | **466** | | **2517** |
| ***Legionella*** | **40** | | | **2** | | **5** |  | **48** | **2** |  | | **97** |
| *Malikia* | 8 | | | 4 | | 39 | 13 | 9 | 17 |  | | 90 |
| *Marinithermus* | 1 | | |  | | 4 | 2 | 5 | 11 | 76 | | 99 |
| *Melissococcus* | 12 | | |  | | 23 | 5 |  | 17 |  | | 57 |

**Supplementary Table S2.** (Continued)

| **Genus** | **Door Buttons** | **Diaper Scale** | **Drawer Handles** | **Key-**  **boards** | **Inside Incubator** | **Sink** | **Touch screen** | **Count Totals** |
| --- | --- | --- | --- | --- | --- | --- | --- | --- |
| *Methylobacterium* |  | 4 | 67 | 3 | 45 | 64 | 26 | 209 |
| *Methylophilus* | 3 |  | 25 | 4 | 19 |  | 29 | 80 |
| ***Microbacterium*** | **31** | **12** | **13** | **5** |  | **12** |  | **73** |
| *Millisia* | 22 | 43 | 69 | 19 | 36 | 519 | 2 | 710 |
| *Mitsuaria* | 2 | 5 | 29 |  | 12 | 2 | 41 | 91 |
| ***Neisseria*** | **14** | **5** | **33** | **3** |  | **39** | **45** | **139** |
| *Niastella* | 78 | 4 | 76 | 5 | 17 | 28 | 51 | 259 |
| *Oligotropha* | 35 | 6 | 21 | 9 | 8 | 8 | 2 | 89 |
| *Paenibacillus* |  |  | 1 | 6 | 7 | 13 | 27 | 54 |
| *Paracoccus* | 7 |  | 6 | 23 |  | 480 | 53 | 569 |
| ***Pasteurella*** | **12** |  | **95** | **8** |  | **24** |  | **139** |
| *Pasteuriaceae* |  | 123 |  | 2 | 8 |  |  | 133 |
| *Paucisalibacillus* |  | 2 |  | 4 | 10 | 1 | 94 | 111 |
| *Polynucleobacter* | 25 |  | 20 | 7 |  |  | 29 | 81 |
| *Prevotella* |  |  | 35 |  |  | 17 |  | 52 |
| ***Propionibacterium*** | **2293** | **265** | **1165** | **780** | **76** | **186** | **350** | **5115** |
| *Propionimicrobium* |  |  |  |  |  | 94 |  | 94 |
| *Proteiniphilum* |  |  |  | 1 |  | 81 |  | 82 |
| ***Pseudomonas*** | **17** | **34** | **19** | **2** | **8** | **4** | **2** | **86** |
| *Pseudonocardia* | 1 | 2 | 12 | 4 | 13 | 12 |  | 44 |
| *Pseudorhodobacter* | 2 |  | 5 | 9 |  | 495 |  | 511 |
| *Reinekea* | 12 | 3 | 19 | 6 | 8 | 6 | 8 | 62 |
| *Rhodobacter* |  | 8 |  |  |  | 15 | 15 | 38 |
| *Rickettsiella* |  |  |  |  | 11 | 6 | 49 | 66 |
| ***Roseomonas*** | **3** | **7** |  | **9** |  | **188** |  | **207** |
| *Salinicoccus* |  |  |  | 18 | 39 | 1 |  | 58 |
| *Sandaracinobacter* | 3782 | 983 | 3694 | 1078 | 1251 | 748 | 4771 | 16307 |
| *Sphingobium* | 37 | 30 | 25 | 20 | 21 | 41 | 39 | 213 |
| *Sphingosinicella* | 77 | 13 | 109 | 22 | 40 | 40 | 47 | 348 |
| ***Staphylococcus*** | **214** | **88** | **436** | **824** | **44** | **58** | **199** | **1863** |
| ***Stenotrophomonas*** | **18** | **154** | **70** | **32** | **8** | **120** | **79** | **481** |
| ***Streptococcus*** | **214** | **165** | **317** | **364** | **40** | **295** | **624** | **2019** |
| *Terrimonas* | 12 | 5 | 5 | 1 | 23 | 2 | 91 | 139 |
| *Thioreductor* |  |  | 42 | 3 | 1 | 5 |  | 51 |
| *Veillonella* | 54 |  | 15 | 26 |  | 18 |  | 113 |
| ***Vibrio*** | **243** | **53** | **196** | **58** | **85** | **59** | **326** | **1020** |
| *Xylella* |  | 5 | 17 |  |  | 6 | 20 | 48 |
| *Xylophilus* | 24 | 10 | 22 | 5 | 46 | 5 | 35 | 147 |
