# Supplementary material for: Bacterial Diversity in Two Neonatal Intensive Care Units (NICUs)
Source: PLoS One. 2013 Jan 23;8(1):e54703. doi: 10.1371/journal.pone.0054703 (PMC3553055; doi:10.1371/journal.pone.0054703)
Supplement: Table S4 — Supplementary Methods. (DOCX) [file pone.0054703.s004.docx]

**Supplementary Methods**

The following studies were retrieved from QIIME database:

| Project Description | STUDY_ID | Citation |
| --- | --- | --- |
| Air samples | 1345 | Kembel (2012) |
| Body Site Samples | 449 | Costello (2009) |
| NICU Samples | 386 | This study |
| Office Samples | 1479 | Hewitt (2012) |
| Soil Samples | 103 | Lauber (2009) |
| Restroom Samples | 1335 | Flores (2011) |
|  | |  |

Analyses were performed using SourceTracker version 0.9.4 and QIIME version 1.5.0-dev (537060a863f6a1e5af2552bf9a4f1476cad4b676)

- export ANALYSIS_DIR=/Users/jc33/Desktop/caporaso_lab/NICU
- filter_samples_from_otu_table.py -m $ANALYSIS_DIR/SourceTrack/map.txt -s 'SourcetrackerEnv:*,!NA' -i $ANALYSIS_DIR/SourceTrack/meta_analysis_tmpaLOHAr1YyAfgga6e0GHv_otu_table_even500.biom -o $ANALYSIS_DIR/SourceTrack/source_track_500_st_only.biom
- filter_otus_from_otu_table.py -i$ANALYSIS_DIR/SourceTrack/source_track_500_st_only.biom -o$ANALYSIS_DIR/SourceTrack/filtered_otu_table_source_track_st_only.biom -s 7
- single_rarefaction.py -i$ANALYSIS_DIR/SourceTrack/filtered_otu_table_source_track_st_only.biom -o $ANALYSIS_DIR/SourceTrack/filtered_otu_table_source_track_st_only_100.biom -d 100
- convert_biom.py -i $ANALYSIS_DIR/SourceTrack/filtered_otu_table_source_track_100.biom -o $ANALYSIS_DIR/SourceTrack/filtered_otu_table_source_track_100.txt -b
- R --slave --vanilla --args -i $ANALYSIS_DIR/SourceTrack/filtered_otu_table_source_track_st_only_100.txt -m $ANALYSIS_DIR/SourceTrack/map-1.txt -o $ANALYSIS_DIR/SourceTrack/sourcetracker_out < /Users/jc33/sourcetracker-0.9.4/sourcetracker_for_qiime.r

Create 3d Biplots

- export ANALYSIS_DIR=/Users/jc33/Desktop/caporaso_lab/NICU
- filter_samples_from_otu_table.py -i meta_analysis_tmpaLOHAr1YyAfgga6e0GHv_otu_table_even500.biom -o otu_table_filtered.biom -m meta_analysis_tmpaLOHAr1YyAfgga6e0GHv_map.txt -s 'PROJECT_NAME:Kelley_office_contamination,Flores_restroom_surface_biogeography,Kelley_Newborn_ICU_Study,Green_hospital_air'
- beta_diversity_through_plots.py -i $ANALYSIS_DIR/tmp1et9ok4nimmeta_analysis_tmpaLOHAr1YyAfgga6e0GHv_map_and_otu_table/otu_table_even_500_filtered.biom -m$ANALYSIS_DIR/tmp1et9ok4nimmeta_analysis_tmpaLOHAr1YyAfgga6e0GHv_map_and_otu_table/meta_analysis_tmpaLOHAr1YyAfgga6e0GHv_map.txt -o nicu_filtered_beta_div -t $ANALYSIS_DIR/gg_otus_4feb2011/trees/gg_97_otus_4feb2011.tre
- summarize_taxa.py -i $ANALYSIS_DIR/NICU_out_files/otu_table_filtered_even_500.biom -o $ANALYSIS_DIR/NICU_out_files/summarize_otu_500_even
- make_3d_plots.py -i $ANALYSIS_DIR/NICU_out_files/nicu_filtered_beta_div/weighted_unifrac_pc.txt -m$ANALYSIS_DIR/tmp1et9ok4nimmeta_analysis_tmpaLOHAr1YyAfgga6e0GHv_map_and_otu_table/meta_analysis_tmpaLOHAr1YyAfgga6e0GHv_map.txt -t $ANALYSIS_DIR/NICU_out_files/summarize_otu_500_even/otu_table_filtered_even_500_L4.txt -o $ANALYSIS_DIR/NICU_out_files/biplot_weight_500_even
